# Acceptance of electronic referrals across the Kingdom of Saudi Arabia: results from a national e-health database

**DOI:** 10.3389/fpubh.2024.1337138

**Published:** 2024-07-17

**Authors:** Abdullah A. Alharbi, Nawfal A. Aljerian, Meshary S. Binhotan, Hani A. Alghamdi, Reem S. AlOmar, Ali K. Alsultan, Mohammed S. Arafat, Abdulrahman Aldhabib, Ahmed I. Aloqayli, Eid B. Alwahbi, Mohammed K. Alabdulaali

**Affiliations:** ^1^Family and Community Medicine Department, Faculty of Medicine, Jazan University, Jazan, Saudi Arabia; ^2^Saudi Medical Appointment and Referrals Center, Ministry of Health, Riyadh, Saudi Arabia; ^3^Emergency Medicine Department, King Saud bin Abdulaziz University for Health Sciences, Riyadh, Saudi Arabia; ^4^King Abdullah International Medical Research Centre, Riyadh, Saudi Arabia; ^5^Emergency Medical Services Department, College of Applied Medical Sciences, King Saud bin Abdulaziz University for Health Sciences, Riyadh, Saudi Arabia; ^6^Department of Family and Community Medicine, College of Medicine, King Saud University, Riyadh, Saudi Arabia; ^7^Department of Family and Community Medicine, College of Medicine, Imam Abdulrahman Bin Faisal University, Dammam, Saudi Arabia; ^8^Ministry of Health, Riyadh, Saudi Arabia

**Keywords:** tele-health, digital health transformation, e-referrals, public health policy, the Saudi Medical Appointments and Referrals Centre, the Kingdom of Saudi Arabia

## Abstract

**Introduction:**

An effective referral system is necessary to ensure quality and an optimum continuum of care. In the Kingdom of Saudi Arabia, an e-referral system known as the Saudi Medical Appointments and Referrals Centre (SMARC), has been fully functioning since 2019. This study aims to explore the rate of medical e-referral request acceptance in the KSA, and to study the factors associated with acceptance.

**Methods:**

This period cross-sectional study utilised secondary collected data from the SMARC e-referral system. The data spans both 2020 and 2021 and covers the entirety of the KSA. Bivariate analyses and binary logistic regression analyses were performed to compute adjusted Odds Ratios (aORs) and 95% confidence intervals.

**Results:**

Of the total 632,763 referral requests across the 2 years, 469,073 requests (74.13%) were accepted. Absence of available machinery was a significant predictor for referral acceptance compared to other reasons. Acceptance was highest for children under 14 with 28,956 (75.48%) and 63,979 (75.48%) accepted referrals, respectively. Patients requiring critical care from all age groups also had the highest acceptance including 6,237 referrals for paediatric intensive care unit (83.54%) and 34,126 referrals for intensive care unit (79.65%). All lifesaving referrals, 42,087 referrals, were accepted (100.00%). Psychiatric patients were observed to have the highest proportion for accepted referrals with 8,170 requests (82.50%) followed by organ transplantations with 1,005 requests (80.92%). Sex was seen to be a significant predictor for referrals, where the odds of acceptances for females increased by 2% compared to their male counterparts (95% CI = 1.01–1.04). Also, proportion of acceptance was highest for the Eastern business unit compared to all other units. External referrals were 32% less likely to be accepted than internal referrals (95% CI = 0.67–0.69).

**Conclusion:**

The current findings indicate that the e-referral system is mostly able to cater to the health services of the most vulnerable of patients. However, there remains areas for health policy improvement, especially in terms of resource allocation.

## Introduction

Healthcare systems face different challenges that influence the provision of access to health services for patients (1). These may include geographical challenges such as access in rural areas, lack of medical equipment and unavailable specialists (1). Linking between healthcare facilities to ensure patients have access to their needed services and to fulfil the gap in health infrastructure is therefore vital (2, 3). A referral system is an integrated system that links different healthcare facilities and regional hospitals together (4). It provides patients with access to specific and needed healthcare resources that are not available at patients’ original sites (5).

The literature indicates a significant variation in referral rates amongst physicians, with some primary care providers issuing referrals at a rate more than five times higher per patient or per visit compared to others (6). The variation is due to several factors including the scope of training and length of experience of physicians, and the healthcare settings (urban vs. rural) (6). These referral requests have also been remarkably increased over the past few years (5, 7). These increases may be attributed to increasing complexity of the required care which subsequently requires more specialised physicians as well as the increasing demands of health care services which is likely due to the growing number of people (5, 8, 9). Subsequently, meeting the increasing number of referral requests is therefore challenging. However, it is critical to provide the required care to these requests as failure to do so may lead to delay in disease diagnosis and proper management.

Despite the overarching intention of a referral system to optimise patient care, not all referrals are accepted. The reasons for rejection can be multifaceted, such as low severity of the case therefore a low priority for referral, multiple referrals for the same person, and limitations in hospital capacity (10, 11). On the other hand, acceptance of referrals implies that the patients’ medical needs align with the receiving hospital’s specialists and available resources. Hence, it is essential to understand the dynamics between acceptance and rejection as it plays an important role in enhancing the quality of healthcare services. It helps to identify areas for improvement to facilitate better coordination of referral processes and to ensure receiving timely and appropriate care.

The Kingdom of Saudi Arabia (KSA) uses a national level electronic referral system known as the Saudi Medical Appointments and Referrals Centre (SMARC). This centre was firstly launched in 2012 and previously known as ‘Ehalati’ (12). However, during the healthcare transformation within the national initiatives for Saudi vision 2030, this centre was expanded to its current formation. The SMARC currently manages and operates all e-referral requests from the 13 administrative regions across the country (5). It provides all registered physicians in the KSA access to the referral system to request referrals when there is a need. The requested referrals are processed in the e-referral system and sent to potential receiving healthcare facilities providing the requested healthcare resources. It also provides a 24-h hotline service where registered physicians can directly call and request lifesaving referrals for critically ill patients, to save time and expedite the referral process. The presence of the system provides an opportunity to epidemiologically explore the patterns of e-referral acceptance across the KSA using the secondary collected data collected by this system. Therefore, this paper aims to examine the level of e-referral request acceptance, and the predictors of these acceptances which will in turn shed light on the effectiveness of the system as well as highlight potential weaknesses and drawbacks worthy of policy changes.

## Materials and methods

### Study design and settings

This period cross-sectional epidemiological study analysed data retrospectively from the Saudi e-referral system known as SMARC. The SMARC system manages all referrals from all 13 administrative regions of the KSA whether internally or externally, i.e., a referral from an institution to another within the same administrative region, or a referral from an institution to another outside of that specific administrative region. This system is unique in that it operates in secondary healthcare systems and above. Therefore, there is no primary care involvement. All data of patients with a referral request initiated between January 2020 and December 2021 have been included in this study.

### Ethical considerations

The institutional review board of the MoH as well as the institutional review board of Imam Abdulrahman Bin Faisal University both approved the study (23-77 E) and (IRB-2023-01-357). The data was completely anonymised with no patient identifying information. Also, the data was properly secured and was used for the purposes of this research.

### Measurements

The data provided by the SMARC e-referral system included basic sociodemographic variables such as age, sex, nationality, and the administrative region from which the patient was referred. These regions were then collated to form the five business units (BUs) that form the basis for the Saudi New Model of Care, namely, the Central BU, Eastern BU, Northern BU, Western BU, and the Southern BU. Also included in the dataset were referral characteristics which included the data of the referral, referral bed type, the type of the referral itself, reason for the referrals, speciality for which the referral is requested, internal vs. external referral, and finally the status of the referral request (accepted vs. rejected). The accepted referral indicates the acceptance of the receiving facility to receive the referred patient whilst the rejected ones indicate the rejection of the receiving facility. The referral status, either accepted or rejected, does not indicate whether the patient attended the referral appointment or not.

### Statistical analyses

The main outcome of this study was whether the referral was accepted or rejected. Descriptive statistics were analysed through frequencies and proportions. Cross tabulations were performed by means of a series of Chi-squared tests. A binary logistic regression analysis was performed to obtain adjusted Odds Ratios (aORs) and their accompanying 95% confidence intervals (CIs). The level of significance was set to 0.05 and the Stata Statistical software version 16 was used for the analyses.

## Results

### Sociodemographic characteristics according to e-referral status

A total of 632,763 referral requests were included in the analyses. Of those, 74.13% were accepted and 25.87% were rejected. Acceptance was highest for children under 14 in general (75.48%), followed by the adult population aged between 25 and 65 years followed by the older people aged above 65 years of age (73.98 and 73.65% respectively). Acceptance was slightly higher for non-Saudis. For seasons, the highest acceptance was during winter and the lowest was during autumn (74.55 and 73.83% respectively). With regards to BUs, the Eastern BU had the highest proportion of acceptance reaching 83.70% whereas the Central BU had the lowest at 69.23%. Internal referral requests were more accepted than external ones (75.54% vs. 68.51%; Table 1).

**Table 1:** Sociodemographic characteristics of patients according to e-referral status between 2020 and 2021 across the Kingdom of Saudi Arabia.

Characteristics	Total	Rejected	Accepted	*p-value*
	*N* (%)	*N* (%)	*N* (%)	
	632,763(100)	163,690 (25.87)	469,073(74.13)	
Age (years)				<0.001^***^
<1	38,361 (6.06)	9,405 (24.52)	28,956 (75.48)	
1–<14	84,762 (13.40)	20,783 (24.52)	63,979 (75.48)	
14–< 18	20,678 (3.27)	5,484 (26.52)	15,194 (73.48)	
18–< 25	48,631 (7.69)	13,160 (27.06)	35,471 (72.94)	
25–65	353,684 (55.90)	92,029 (26.02)	261,655 (73.98)	
>65	86,647 (13.69)	22,829 (26.35)	63,818 (73.65)	
Sex				*0.129*
Males	341,059 (53.90)	89,493 (26.24)	251,565 (73.76)	
Females	291,703 (46.10)	76,017 (26.06)	215,685 (73.94)	
Nationality				0.006^**^
Non-Saudi	88,215 (13.94)	22,493 (25.50)	65,722 (74.50)	
Saudi	544,548 (86.06)	141,197 (25.93)	403,351 (74.07)	
Year				<0.001^***^
2020	275,956 (43.61)	73,453 (26.62)	202,503 (73.38)	
2021	356,807 (56.39)	90,237 (25.29)	266,570 (74.71)	
Seasons				<0.001^***^
Winter	173,751 (27.46)	44,224 (25.45)	129,527 (74.55)	
Spring	127,968 (20.22)	33,427 (26.12)	94,541 (73.88)	
Summer	153,950 (24.33)	39,685 (25.78)	114,265 (74.22)	
Autumn	177,094 (27.99)	46,354 (26.17)	130,740 (73.83)	
Business units				<0.001^***^
Central	96,778 (15.29)	29,778 (30.77)	67,000 (69.23)	
Eastern	70,461 (11.14)	11,487 (16.30)	58,974 (83.70)	
Western	222,151 (35.11)	55,314 (24.90)	166,837 (75.10)	
Northern	113,011 (17.86)	29,799 (26.37)	83,212 (73.63)	
Southern	130,362 (20.60)	37,312 (28.62)	93,050 (71.38)	
External vs. internal				<0.001^***^
Internal	505,650 (79.91)	123,660 (24.46)	381,990 (75.54)	
External	127,113 (20.09)	40,030 (31.49)	87,083 (68.51)	

Results were presented as frequency [number (*N*) and percent (%)]. The relationship between variables was assessed using the chi-square test. ^*^Significant difference at *p* ≤ 0.05, ^**^significant difference at *p* ≤ 0.01, and ^***^significant difference at *p* ≤ 0.001.

### E-referral characteristics according to the e-referral status

Table 2 shows the bivariate associations between e-referral characteristics and the status of referral requests. Patients who require paediatric intensive care unit (PICU) beds had the highest acceptance, followed by patients who required intensive care unit beds (ICU) (83.54% and 79.65% respectively). Patients who required a regular ward bed had the lowest acceptance (72.13%). According to referral types, life-saving requests were all accepted (100.00%). As for the reason for referral, health crisis had the highest referral acceptance, followed by the unavailability of a specialised physician (75.86% and 75.17%). Patients with social reasons had the lowest acceptance (69.80%).

**Table 2:** E-referral characteristics according to the status of e-referral requests between 2020 and 2021 across the Kingdom of Saudi Arabia.

Referral characteristics	Total	Rejected	Accepted	*p*-value
	*N* (%)	*N* (%)	*N* (%)	
	632,763(100)	163,690 (25.87)	469,073(74.13)	
Bed types				<0.001^***^
OPD no bed	302,815 (47.86)	78,256 (25.84)	224,559 (74.16)	
Ward	225,600 (35.65)	62,885 (27.87)	162,715 (72.13)	
Burn bed	552 (0.09)	131 (23.73)	421 (76.27)	
Isolation	25,423 (4.02)	6,156 (24.21)	19,267 (75.79)	
CCU Bed	17,560 (2.78)	3,978 (22.65)	13,582 (77.35)	
NICU	10,501 (1.66)	2,335 (22.24)	8,166 (77.76)	
ICU	42,846 (6.77)	8,720 (20.35)	34,126 (79.65)	
PICU	7,466 (1.18)	1,229 (16.46)	6,237 (83.54)	
Referral types				<0.001^***^
Life saving	42,087 (6.65)	0(0.00)	42,087 (100.00)	
Routine OPD	304,188 (48.07)	78,641(25.85)	225,547 (74.15)	
Routine admission	81,899 (12.94)	20,751(25.34)	61,148 (74.66)	
ER	204,589 (32.33)	64,298(31.43)	140,291 (68.57)	
Reason for referral				<0.001^***^
Unavailable subspecialty	390,054 (61.64)	101,509 (26.02)	288,545 (73.98)	
Unavailable physician	108,012 (17.07)	26,815 (24.83)	81,197 (75.17)	
Unavailable machine	84,931 (13.42)	22,162 (26.09)	62,769 (73.91)	
Unavailable Bed	22,465 (3.55)	6,519 (29.02)	15,946 (70.98)	
Social	1,573 (0.25)	475 (30.20)	1,098 (69.80)	
Health crisis	25,728 (4.07)	6,210 (24.14)	19,518 (75.86)	

Results were presented as frequency [number (*N*) and percent (%)]. The relationship between variables was assessed using the chi-square test. ^***^Significant difference at *p* ≤ 0.001. OPD, Outpatient Department; CCU, Coronary Care Unit Bed; NICU, Neonatal Intensive Care Unit; ICU, Intensive Care Unit; PICU, paediatric Intensive Care Unit; ER, Emergency Room.

### Distribution of medical specialities according to referral status

**Figure 1:**
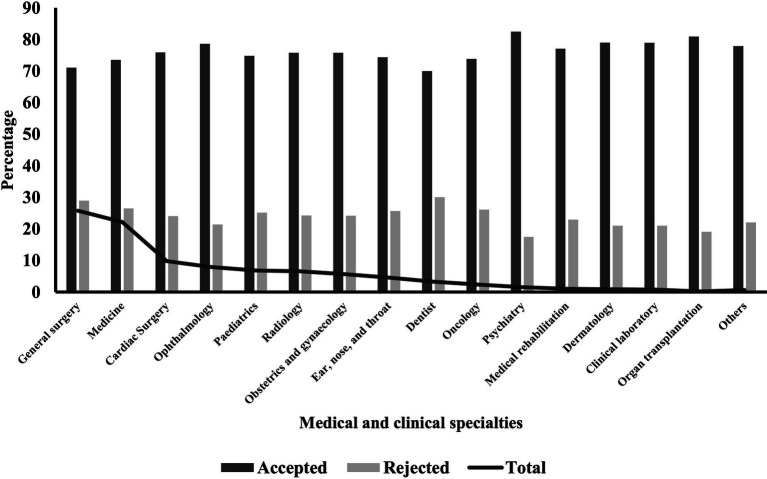
Distribution of medical specialities according to referral status.

According to the distribution of specialties in Table 3 and Figure 1, general surgery had initiated the highest proportion of referral requests followed by medicine (25.77% and 22.18% respectively). With regards to referral status, psychiatry had the highest acceptance reaching 82.50% followed by organ transplantation (80.92%). Dentistry had the lowest acceptance at 69.96%. Differences were significant at the <0.001 level.

**Table 3:** Distribution of medical and clinical specialties according to referral status between 2020 and 2021 across the Kingdom of Saudi Arabia.

Medical and clinical specialties^¥^	Total	Rejected	Accepted
	*N* (%)	*N* (%)	*N* (%)
	632,763(100)	163,690 (25.87)	469,073(74.13)
General surgery	163,052 (25.77)	47,172(28.93)	115,880(71.07)
Medicine	140,334 (22.18)	37,187(26.50)	103,147(73.50)
Cardiac Surgery	61,836 (9.77)	14,876(24.06)	46,960(75.94)
Ophthalmology	50,218 (7.94)	10,739(21.38)	39,479(78.62)
Paediatrics	43,168 (6.82)	10,863(25.16)	32,305(74.84)
Radiology	41,673 (6.59)	10,103(24.24)	31,570(75.76)
Obstetrics and gynaecology	35,831(5.66)	8,668(24.19)	27,163(75.81)
Ear, nose, and throat	28,919 (4.57)	7,416(25.64)	21,503(74.36)
Dentist	20,799 (3.29)	6,247(30.04)	14,552(69.96)
Oncology	14,847 (2.35)	3,882(26.15)	10,965(73.85)
Psychiatry	9,903(1.57)	1733(17.50)	8,170(82.50)
Medical rehabilitation	6,534(1.03)	1,498(22.93)	5,036(77.07)
Dermatology	5,598 (0.88)	1,176(21.01)	4,422(78.99)
Clinical laboratory	4,960 (0.78)	1,044(21.05)	3,916(78.95)
Organ transplantation	1,242(0.20)	237(19.08)	1,005(80.92)
Others	3,849(0.61)	849(22.06)	3,000(77.94)
*P-*value < 0.001^***^			

Results were presented as frequency [number (*N*) and percent (%)]. The relationship between variables was assessed using the chi-square test. ^***^Significant difference at *p* ≤ 0.001.

### Distribution of BUs and administrative regions of the KSA according to referral status

**Figure 2:**
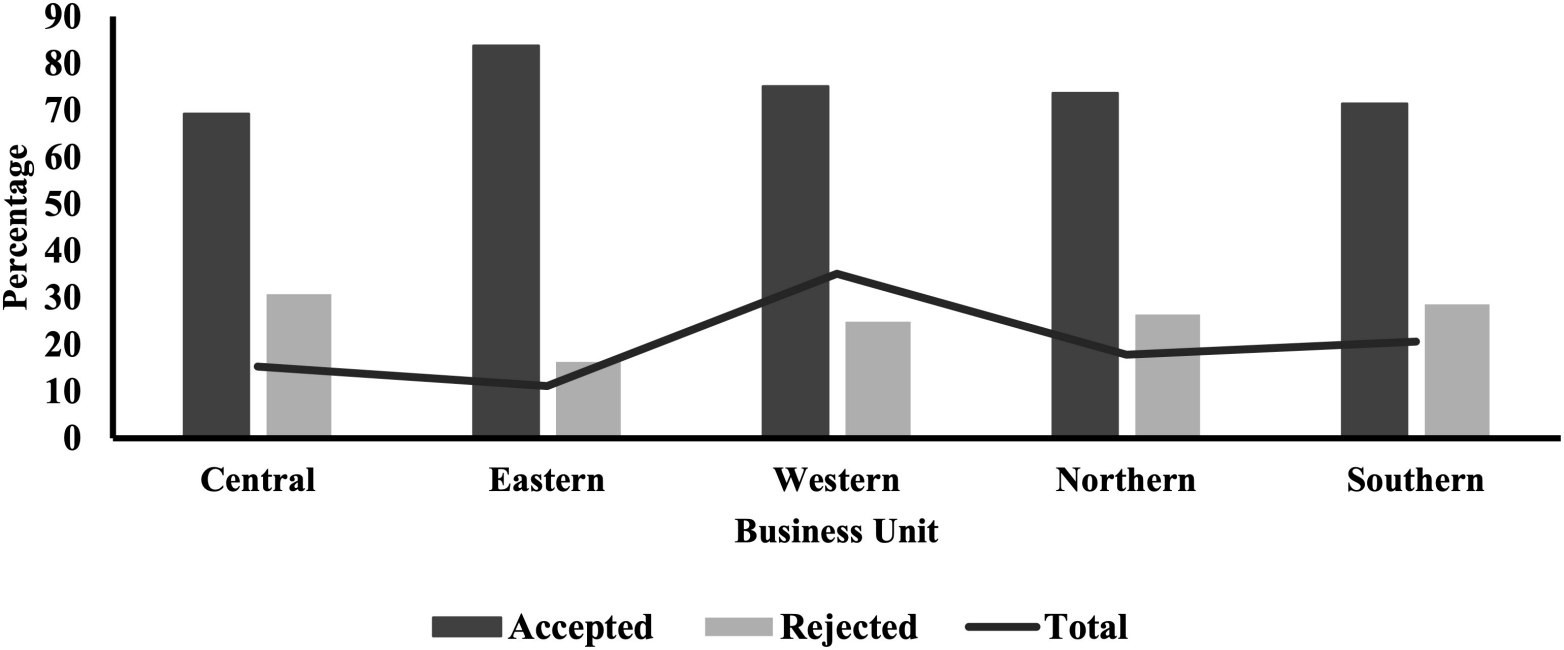
Referral status across the five business units of the Kingdom of Saudi Arabia.

**Figure 3:**
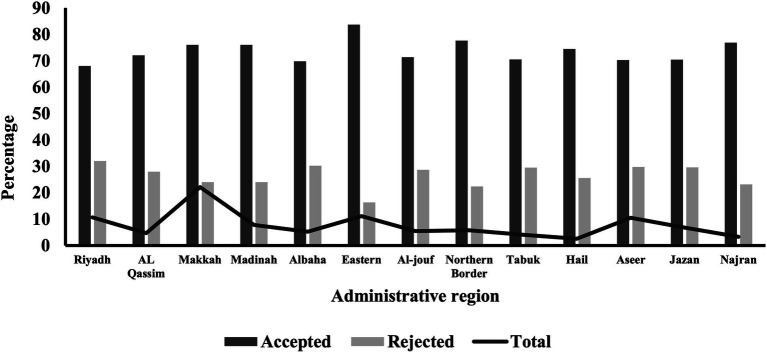
Referral status across 13 administrative regions of the Kingdom of Saudi Arabia.

The distribution of BUs and administrative regions in the KSA showed variations in terms of initiating referral requests and their acceptance rates, as illustrated in Table 4. The Western BU initiated the highest proportion of referral requests (35.11%), followed by the Southern BU (20.60%). Regarding referral acceptance, the Eastern BU had the highest rate at 83.70%, followed by the Western BU at 75.10% (Figure 2). Amongst the 13 administrative regions, Makkah initiated the highest proportion of referral requests (22.12%). The Eastern region had the highest acceptance rate (83.70%), whilst Riyadh had the lowest (68.01%; Figure 3). The differences in referral acceptance rates across BUs and administrative regions were statistically significant (*p* < 0.001).

**Table 4:** Referrals according to referral status across the five business units and 13 administrative regions of the Kingdom of Saudi Arabia.

Business units		Total	Rejected	Accepted
		*N* (%)	*N* (%)	*N* (%)
		632,763(100)	163,690 (25.87)	469,073(74.13)
Central		96,778(15.29)	29,778(30.77)	67,000(69.23)
Eastern		70,461(11.14)	11,487(16.30)	58,974(83.70)
Western		222,151(35.11)	55,314(24.90)	166,837(75.10)
Northern		113,011(17.86)	29,799(26.37)	83,212(73.63)
Southern		130,362(20.60)	37,312(28.62)	93,050(71.38)
*P-*value < 0.001^***^				
**Business units**	**Administrative regions**	**Total**	**Rejected**	**Accepted**
		***N* (%)**	***N* (%)**	***N* (%)**
		632,763(100)	163,690(25.87)	469,073(74.13)
Central	Riyadh	67,203(10.62)	21,499(31.99)	45,704(68.01)
	AL Qassim	29,575(4.67)	8,279(27.99)	21,296(72.01)
Western	Makkah	139,986(22.12)	33,569(23.98)	106,417(76.02)
	Madinah	49,300(7.79)	11,826(23.99)	37,474(76.01)
	Albaha	32,865(5.19)	9,919(30.18)	22,946(69.82)
Eastern	Eastern	70,461(11.14)	11,487(16.30)	58,974(83.70)
Northern	Al-jouf	34,512 (5.45)	9,891(28.66)	24,621(71.34)
	Northern Border	36,707(5.80)	8,221(22.40)	28,486(77.60)
	Tabuk	25,604(4.05)	7,547(29.48)	18,057(70.52)
	Hail	16,188(2.56)	4,140(25.57)	12,048(74.43)
Southern	Aseer	66,458(10.50)	19,752(29.72)	46,706(70.28)
	Jazan	42,953(6.79)	12,704(29.58)	30,249(70.42)
	Najran	20,951(3.31)	4,856(23.18)	16,095(76.82)
*P-*value < 0.001^***^				

Results were presented as frequency [number (*N*) and percent (%)]. The relationship between variables was assessed using the chi-square test. ^***^Significant difference at *p* ≤ 0.001.

### Multivariable associations of predictors for referral acceptance

Table 5 shows the adjusted multivariable associations of predictors with referral acceptance. Children under 14 years of age have exhibited a statistically significant increased likelihood of referral acceptance when compared to adults aged 25 to 65 years old (aOR = 1.05, 95% CI = 1.02–1.08 and aOR = 1.09, 95% CI = 1.07–1.12). Conversely, the older people aged above 65 years of age had decreased likelihood of acceptance (aOR = 0.96, 95% CI = 0.94–0.97). Females were 2% more likely to have their requests accepted compared to males (95% CI = 1.01–1.04). Also, the data shows that there is a 6% increase in referral acceptance in 2021 compared to 2021 (95% CI = 1.05–1.08). The winter season had a significantly higher likelihood of referral acceptance when compared to spring (aOR = 1.06, 95% CI = 1.04–1.08). With regards to the BUs, the Eastern BU had more than double the likelihood of referral acceptance (aOR = 2.39, 95% CI = 2.33–2.45). Higher acceptance was also more likely in all other BUs compared to the Central BUs, although less extremely. When comparing bed types to ward beds, requests involving PICU were 93% more likely to be accepted (95% CI = 1.80–2.06). followed by ICU requests (aOR = 1.59, 95% CI = 1.55–1.64). All other bed types were also more likely to be accepted compared to regular ward beds except for burn beds. Upon examination of reasons for referral, requests with a reason of an unavailability of speciality or specialised physicians, unavailability of a bed, and social reasons were statistically significantly less likely to be accepted compared to requests with a reason of an unavailability of machine. Also, external referrals were 32% less likely to be accepted compared to internal referrals (95% CI = 0.67–0.69).

**Table 5:** Multivariable logistic regression analysis of predictors for referral acceptance between 2020 and 2021 across the Kingdom of Saudi Arabia.

Predictors	Adjusted OR	*p*-value	95% CI
**Age**			
<1	1.05	0.001^***^	1.02–1.08
1–<14	1.09	<0.001^***^	1.07–1.12
14–< 18	0.99	*0.78*	0.96–1.03
18–< 25	0.98	*0.16*	0.96–1.01
25–65	*Ref*		
>65	0.96	0.001^***^	0.94–0.97
**Sex**			
Males	*Ref*		
Females	1.02	<0.001^***^	1.01–1.04
**Nationality**			
Non-Saudi	*Ref*		
Saudi	0.99	*0.789*	0.979–1.017
**Year**			
2020	*Ref*		
2021	1.06	<0.001^***^	1.05–1.08
**Season**			
Spring	*Ref*		
Winter	1.06	<0.001^***^	1.04–1.08
Summer	1.01	*0.13*	0.99–1.03
Autumn	1.01	*0.15*	0.99–1.03
**Business units**			
Central	*Ref*		
Eastern	2.39	<0.001^***^	2.33–2.45
Western	1.43	<0.001^***^	1.40–1.45
Northern	1.45	<0.001^***^	1.42–1.48
Southern	1.17	<0.001^***^	1.15–1.19
**Bed types**			
Ward beds	*Ref*		
OPD	1.10	<0.001^***^	1.08–1.11
Burn beds	1.27	0.02^*^	1.03–1.57
Isolation beds	1.18	<0.001^***^	1.14–1.22
CCU	1.40	<0.001^***^	1.34–1.45
NICU	1.44	<0.001^***^	1.36–1.51
ICU	1.59	<0.001^***^	1.55–1.64
PICU	1.93	<0.001^***^	1.80–2.06
**Reason for referral**			
Unavailable machine	*Ref*		
Unavailable subspecialty	0.93	<0.001^***^	0.91–0.95
Unavailable physician	0.98	*0.089*	0.96–1.00
Unavailable bed	0.73	<0.001^***^	0.70–0.76
Social reasons	0.61	<0.001^***^	0.55–0.69
Health crisis	0.96	*0.067*	0.93–1.00
**Referral direction**			
Internal	*Ref*		
External	0.68	<0.001^***^	0.67–0.69

^*^Significant difference at *p* ≤ 0.05, ^**^Significant difference at *p* ≤ 0.01, and ^***^significant difference at *p* ≤ 0.00.

## Discussion

This study aimed to explore the proportion of accepted e-referral requests and identify their predictors using national-level secondary data of over 600,000 requests across the KSA. No prior research has examined e-referral acceptance on such a large, nationwide scale. The study found that the overall acceptance rate for referral requests was 74.13%. Key predictors that increased the likelihood of acceptance included children under 14, the absence of available machinery, patients requiring critical care, psychiatric patients and those needing organ transplants, being female, and referrals between facilities within the same region (internal referrals). On the other hand, lower acceptance was associated with older adults, males, regular ward bed requests, requests with reasons like unavailability of a specialty/physician or social reasons, and external referrals. The findings provide comprehensive insights into e-referral acceptance and rejection patterns across various key factors, offering valuable information to optimise healthcare delivery and resource allocation.

### E-referral acceptance according to sociodemographic characteristics

Around three-fourths of all referrals were accepted. This proportion of acceptance highlights the critical need to provide the necessary care for these cases. It suggests the healthcare system was generally able to accommodate the majority of referred patients. The analysis also showed that acceptance proportions were quite similar across the different age groups. However, compared to the 25–65-year-old age group, children under 14 years old, including neonates and infants, were more likely to have their referrals accepted. This may reflect the special healthcare needs that are time-sensitive due to the critical nature of their paediatric condition, especially since that for neonates and infants, fragility and rapid deterioration due to the low immunity may render their medical situation unpredictable (13, 14). Also, it may be due to the availability of highly specialised maternal and children hospitals in large referral regions which in turn facilitates a higher proportion of acceptance compared to other age groups (15).

Referrals by sex showed an overall higher referral request for males but a higher proportion of acceptance for females. Sex-specific health conditions, such as prenatal care, gynaecological conditions, and breast health could provide an explanation to the increased accepted referrals amongst females compared to males. For example, obstetric complications preceding delivery could be life-threatening and necessitate specialised care services that may be lacking in the same region which consequently would make it more probable that such a referral by accepted (16). Additionally, specialised maternity and children hospitals in the different regions in the KSA might expedite the process of acceptance for females (15).

Similarly, referrals by nationality exhibited higher referrals for Saudis but slightly less rejected requests for expatriates. Though non-Saudis are medically covered by private insurance, initiated referrals could be due to the need for specialist care that is not available in the private sector or not covered by their insurance (17, 18).

### E-referral acceptance according to e-referral characteristics

The current analysis shows that PICU referrals were fewer in number but had the highest proportion of acceptance. All other critical care bed types including NICU, ICU and CCU were also amongst the highest for acceptance, which shows a higher prioritisation for the critically ill. Critically ill patients are in need for close monitoring and management of these patients are time sensitive (19). Several studies have showed that delay in treating critically ill patients can contribute to increasing complications and mortality (19–23). The fact that these cases are usually accepted may also indicate the lack of specialised services or specialised staff to deal with these cases in the places of referral request initiation.

With regards to the type of referrals, it is of importance to note that all referrals that were tagged or entered as ‘lifesaving’ were accepted (100.00%). This shows that the system is functioning according to its objectives in terms of catering for the most needed and more vulnerable of cases. Whereas for emergency referrals, around two-third of them were accepted. This lower acceptance of emergency cases raises the questions as to whether they were genuinely emergency cases or were in fact not emergent and were simply due to a lack of resources and capabilities. It also raises the question of how those patients will receive the necessary treatment in the event of a refusal.

Upon examination of the reasons for referrals as a predictor for acceptance, a major predictor was the lack of machine or equipment. Equipment availability is a global issue, and such complex equipment requires maintenance, training of qualified users, then replacement (24–26). Consequently, some advanced medical technologies might not be feasible to be available in every healthcare institution thereby naturally requiring a referral of patients. However, medical equipment is a key in healthcare services as it is routinely used for diagnosis, treatment, and rehabilitation (27, 28). Thus, they are significant in providing the care needed for patients to improve and recover (29). In some cases, lack of access to medical equipment can result in poor prognosis and mortality (30, 31).

The current analyses show that psychiatry was the speciality with the highest proportion of acceptance. The KSA currently has 19 specialised psychiatric complexes and hospitals distributed across the 13 administrative regions (32). The high availability of resources needed to treat psychiatric patients may have contributed towards the high proportion of acceptance. Similarly, there are 11 centres for organ transplantations across the KSA (33). These centres are distributed in the five BUs with the Western and Central units as the highest, which could be explained by the high population density at these two units (34). A further explanation could be that organ transplantation is a successful method to improve morbidity and mortality, but only if managed swiftly (35, 36). The time sensitivity in organ transplantation might explain the existence of a special pathway for it in the KSA to expedite the process (37). All these factors may have allowed for the high proportion of acceptance for organ transplantations. On the other hand, dental services were found to be amongst the lowest in terms of referral request initiation, but also is the speciality with the lowest proportion of acceptance. This may be due to the low utilisation of dental services in the country, where literature shows that only 11.5% of Saudi adults visit dentists for routine checkups but may also be due to the low prioritisation of dental care services provided (38).

Variation between BUs was noticed in terms of the total referrals and the proportion of acceptance (Table 4). All BUs demonstrated a higher odd of acceptance than the Central BU. Despite the fact that the lowest referral requests originated from the Eastern BU, they were the highest accepted amongst all BUs. The Eastern BU was able to successfully implement the New Model of Care that yielded positive results including care provision and disease prevention (39). In contrary, referrals originating from the Central BU had the highest proportion of rejections. Hence, there is a need to explore the reasons of referrals in the central BUs that could explain the proportion of rejection. It is worth mentioning that the higher density of the population in the Central region, along with the easy access to private healthcare services, might add explain the higher rejections. The high socioeconomic status amongst people living in Riyadh might opt for out-of-pocket payments to access private care services instead of awaiting approval within governmental hospitals or after their referral requests have been rejected (40).

Finally, the trend of referrals had noticeably increased in 2021 in comparison to the preceding year which could be attributable to different factors. First, the expansion of healthcare providers within the network may have led to more referrals being initiated. Second, governmental support to strengthen the SMARC e-referral system could have positively influenced the referral process. This support has the potential to improve the efficiency and adoption of the SMARC system. Third, software improvement and enhancement could have streamlined and facilitated the referral process. Finally, increased awareness and trust on the SMARC system amongst healthcare providers from different specialties could have played an important role in the higher utilisation in 2021. It is worth mentioning that despite the challenges posed by the COVID-19 pandemic, the proportion of accepted referrals in 2021 was higher than the previous year. This could be due to the measures taken to adapt to the pandemic and to improve the delivery of healthcare system accordingly.

Nevertheless, the COVID-19 pandemic had a significant impact on healthcare services worldwide, and the KSA was no exception, particularly in managing chronic diseases and maintaining essential healthcare services (41). The sudden and global spread of COVID-19 led to unprecedented changes in healthcare systems and shifted the focus of healthcare providers to manage the pandemic (42). Whilst COVID-19 may have influenced the volume of healthcare referrals in the KSA, it is not possible to make a direct comparison as referral patterns prior to the pandemic were not investigated. Despite this, many studies have shown that the pandemic had a significant impact on healthcare delivery worldwide, including referral rates (43–46). The prevalence of COVID-19 has significantly affected the referral and admission rates for non-COVID-19 patients due to health measures like self-isolation, quarantine, and stay-at-home recommendations, as well as the fear of infection in medical centres (46). Our data have shown that the number of healthcare referrals decreased in 2020 compared to 2021, but the acceptance rate was higher in 2021. This trend could be attributed to the severity of the pandemic during the early months of 2020. In the future, longitudinal studies should focus on the impact of the pandemic specifically on the referral system during and after the pandemic to investigate its real effect. This will help to improve preparedness for critical periods in healthcare delivery.

### Recommendations

According to the findings of this study, several key recommendations can be proposed to address identified disparities and improve the efficiency of e-referrals in the KSA. Given the variation in e-referrals by age, targeted interventions can be designed and tailored specifically for people at high risk. This can include proactive management of the prevailing conditions and preventive care strategies, which in return can reduce the need for referrals. Regional variation highlights the need for region-specific interventions to enhance healthcare infrastructure and streamline referrals. This can include increasing the availability of outpatient department, ward beds and medical technologies to meet the high demand and ensure timely and appropriate care. In addition, the effective prioritisation system for critical care referrals, including life-saving and ICU, necessitates ensuring the continuous availability of resources to maintain the high acceptance rate. It is also recommended to address the gap in human resources given the top reason for referrals due to unavailable subspecialties by investing in training and recruitment.

Further research should also investigate the impact of COVID-19 during various pandemic peaks and across different seasons on the efficiency of the ICU referral system. This study would provide valuable insights into how the referral system adapted and responded under the pressures of fluctuating patient volumes and changing healthcare needs during the pandemic. Additional research is needed to assess the effect on general healthcare services provided by the Saudi Medical Referral Centre (SMARC) during these periods. Understanding the broader implications of the pandemic on general healthcare services will help in identifying strengths and potential areas for improvement in the healthcare system’s response to public health emergencies.

### Strength and limitations

This study is the first to investigate the e-referral status in terms of acceptance vs. rejection and its potential predictors on a national level. This novel study highlights the significance of having such a national level system to monitor patients’ access to healthcare services, and subsequently plan for improvement. This study specifically provides a comprehensive insight on the allocation of healthcare resources in all regions across the KSA, which supports policy makers to make well-studied decisions. It also analysed data for two consecutive years which allows measuring the overall trend of referral requests. However, this study has some limitations that need to be acknowledged. First, the retrospective design used in this study limits the ability to establish a relationship about the predictors/factors of referral request acceptance. Second, due to the uniqueness of the data and the system itself, there are limitations for comparison with existing literature. Third, the effects of COVID-19 on the e-referral system and the kind of referral demands, especially during 2020 and 2021, should not be minimised.

## Conclusion

Acceptance of referral requests was predominant in the current practice of e-referrals across the KSA. This study analysed all e-referral requests initiated from secondary healthcare systems and above over 2 years. The findings indicate that the majority of the requests were accepted, with all lifesaving referrals being accepted. Acceptance rates were highest for children under 14, female patients, critical care patients, including those in the PICU, ICU, and CCU, psychiatric patients, organ transplant recipients, and those referred from the Eastern BUs. These results illustrate the importance of prioritising patient care and health patient populations, especially those with life-saving and critical conditions. The study highlights the need for resource allocation assessment to reduce referral requests and improve acceptance and access to care.

## Data availability statement

The datasets presented in this article belong to the Saudi Medical Appointments and Referrals Centre (SMARC) and are not publicly available. Researchers interested in accessing the data should direct their requests to the first author of this article, [AAlh, aaalharbi@jazanu.edu.sa], who will facilitate obtaining permission from SMARC. Access will only be granted after SMARC reviews and approves each request.

## Author contributions

AAlh: Conceptualization, Data curation, Formal analysis, Investigation, Software, Writing – original draft, Writing – review & editing. NA: Conceptualization, Project administration, Resources, Supervision, Writing – review & editing. MB: Data curation, Investigation, Methodology, Software, Writing – original draft. HA: Data curation, Investigation, Methodology, Software, Writing – original draft. RA: Formal analysis, Investigation, Methodology, Software, Writing – original draft. AAls: Conceptualization, Resources, Supervision, Validation, Writing – review & editing. MAr: Conceptualization, Formal analysis, Methodology, Resources, Writing – review & editing. AAld: Conceptualization, Project administration, Supervision, Writing – review & editing. AAlo: Investigation, Project administration, Software, Writing – review & editing. EA: Data curation, Project administration, Resources, Supervision, Writing – review & editing. MAl: Methodology, Resources, Supervision, Writing – review & editing.
